# Impact of Consuming ‘Toxic’ Monarch Caterpillars on Adult Chinese Mantid Mass Gain and Fecundity

**DOI:** 10.3390/insects8010023

**Published:** 2017-02-17

**Authors:** Jamie L. Rafter, Liahna Gonda-King, Daniel Niesen, Navindra P. Seeram, Chad M. Rigsby, Evan L. Preisser

**Affiliations:** 1 Department of Biological Sciences, University of Rhode Island, Kingston, RI 02881, USA; lmgondaking@gmail.com (L.G.-K.); chad3332@gmail.com (C.M.R.); preisser@uri.edu (E.L.P.); 2 Department of Biology, Muskingum University, New Concord, OH 43762, USA; 3 Department of Biomedical and Pharmaceutical Sciences, University of Rhode Island, Kingston, RI 02881, USA; dan_niesen@my.uri.edu (D.N.); nseeram@uri.edu (N.P.S.)

**Keywords:** *Tenodera sinensis*, *Danaus plexippus*, fecundity, monarch, prey toxicity

## Abstract

Predators that feed on chemically-defended prey often experience non-lethal effects that result in learned avoidance of the prey species. Some predators are able to consume toxic prey without ill-effect. The Chinese mantid is able to consume cardenolide-containing monarch caterpillars without immediate adverse effects. Although they discard the caterpillars’ gut contents, mantids consume sequestered cardenolides. Although consumption of these cardenolides does not elicit an acute response, there may be long-term costs associated with cardenolide consumption. We tested the hypothesis that consumption of monarch caterpillars will adversely affect adult mantid biomass gain and reproductive condition. We reared mantids from egg to adult and assigned them to one of four toxicity groups that differed in the number of monarch caterpillars offered over a 15-day period. Mantids consumed similar amounts of prey biomass during the experiment. Yet, mantids in the high-toxicity group had a higher conversion efficiency and gained more biomass than mantids in other groups. Mantids in all treatment groups produced similar numbers of eggs. However, mantids in the high-toxicity group produced heavier eggs and devoted a greater portion of their biomass toward egg production than those in the control group. This increase in reproductive condition is probably driven by variation in prey nutritional value and/or the nutritional advantages inherent in eating multiple food types. Our results demonstrate the mantid is able to incorporate ‘toxic’ monarch prey into its diet without acute or chronic ill-effects.

## 1. Introduction

Chemically-defended species often advertise their protection via aposematism [1,2,3]. These defenses generally involve compounds that are bitter tasting and cause vomiting or other ill effects shortly after consumption. These adverse but non-lethal effects allow predators to learn to avoid consumption of chemically-defended prey [4]. These defenses are not always effective, however, and some predators feed on chemically-defended prey without any immediate ill-effects. The ladybird beetle *Harmonia axyridis*, for example, can metabolize toxic alkaloids produced by the coccinellid species on which it feeds [5]. The harvestman *Mitopus morio* feeds on the larvae of the leaf beetle, *Oreina cacaliae*, and is similarly able to prevent bioactivation and detoxify the toxic pyrrolizidine alkaloids sequestered by the prey [6]. Even predators that lack physiological adaptations can avoid or limit their exposure to prey defenses by processing their prey [7,8,9] or limiting their consumption [10].

Even when predators are able to consume toxic prey with seemingly little effect, there may still be fitness costs associated with toxin consumption. When orb web spiders, *Zygiella x-notata*, feed on oleander aphids, *Aphis nerii*, they suffer disorientation and begin to construct webs that are less efficient at prey capture [11]. The two-spotted ladybird beetle, *Adalia bipunctata*, suffers lower fecundity and egg viability when consuming aphids reared on high-glucosinolate plants [12].

The Chinese mantid, *Tenodera sinensis*, is a generalist predator that has been repeatedly observed feeding on chemically-defended monarch caterpillars, *Danaus plexippus*, in the field with no apparent ill effect [13]. Monarch caterpillars feed on host plants in the genus *Asclepias* (Apocynaceae) that contain cardenolides; the larvae sequester these cardenolides in their bodies, rendering them unpalatable to many predators [14]. We have previously found [15,16] that mantids discard the gut tissue from monarch larvae while consuming the rest of the caterpillar. This gutting behavior does not, however, prevent mantids from consuming cardenolides: while monarch gut and body tissue differed in cardenolide composition, they contained similar cardenolide concentrations [15]. Though mantids suffer no immediate ill-effects from consuming monarch larvae, their consumption of this cardenolide-containing tissue may nonetheless have long-term impacts. Juvenile mantids feeding on a diet containing ground milkweed bugs, *Oncopeltus fasciatus*, exibit decreased consumption and therefore decreased growth efficiencies [17]. Additionally, juvenile mantids fed live milkweed bugs and palatable prey (fruit flies) also reduce consumption of toxic prey resulting in similar reductions in growth and development [18]. We tested whether consuming cardenolide-containing monarch caterpillars reduces adult mantid mass gain and fecundity.

## 2. Materials and Methods

### 2.1. Insect Rearing and Maintenance

We collected a *Tenodera sinensis* egg mass in mid-January 2013 from East Farm (Kingston, RI, USA), an abandoned agricultural field. We placed it in a 50 × 25 × 30 cm Plexiglas aquarium that was kept in a Percival growth chamber with a 16:8 L:D photoperiod and 60%–80% humidity at 25 °C during lighted hours and 23 °C during dark hours until the eggs began to hatch. After hatching, 105 nymphs were placed in individual 1.9 L mason jars, with mosquito netting used in lieu of the tops for ventilation. Because they emerged from a single egg mass, all nymphs were either full- or half-sibs; using related individuals in controlled experiments is a commonly-used means for minimizing the magnitude of uncontrolled population-level variation [19]. A mesh strip was secured under the lid to serve as a perching site and water wicks were made using capped soufflé cups with braided dental cotton inserted through a hole in the lid. These jars were kept in the Percival growth chamber. Mantids in their first four instars were fed lab-reared apterous fruit flies, *Drosophila melanogaster*, purchased from Carolina Biological (Burlington, NC, USA). After mantids reached the fourth instar, they were fed two appropriately-sized crickets daily. Just prior to and during molting, mantids are vulnerable to cricket predation; to prevent this, we tested for satiation by using forceps to offer each mantid a cricket before placing crickets into the jars. If the mantid refused to attack the cricket we assumed it was preparing to molt and did not feed it that day. To help deter crickets from attacking the mantids, we also put fruit flies into the jars for the crickets to eat.

Monarch eggs were purchased from Flutterby Gardens (Bradenton, FL, USA) and reared in the lab on *Asclepias curassavica*, a milkweed species that contains high cardenolide concentrations [20]. Host plants were grown from seed in the University of Rhode Island greenhouse.

### 2.2. Experimental Design

Once mantids reached adulthood, 31 virgin females were randomly assigned to one of four treatments: non-toxic control, low toxicity, medium toxicity, and high toxicity (Table 1). After being assigned to their treatment, all mantids were held for three days without food. As outlined in Table 1, toxicity treatments were determined by the number of fifth-instar monarchs (0, 1, 5, or 15; weighing 0.94 ± 0.022 g [mean (SE)]) offered to a given mantid over a 15-day period (days 4–18). On days during the 15-day treatment period when a mantid was not offered a monarch caterpillar, two crickets (comparable in weight to a single late-instar monarch caterpillar) were offered to the mantid as non-toxic prey. The offering of crickets on non-monarch days was necessary to prevent mantid starvation in the control (zero caterpillars), low-toxicity (one caterpillar), and medium-toxicity (five caterpillars) treatments. If mantids refused to eat a monarch caterpillar, we continued to offer a caterpillar on subsequent days until the mantid accepted the prey; we did not offer mantids crickets unless they had already accepted the caterpillar. Following the 15-day treatment period, all mantids were fed two crickets daily until day 35; this step was necessary in order to give all of the mantids sufficient resources to produce egg masses. We recorded mantid weight before and after feeding as well as prey weight to determine prey biomass consumed. On day 35 mantids were weighed and anesthetized using a kill jar containing ethyl acetate. We dissected each mantid, removed and weighed the egg mass, counted the eggs, and determined the average egg weight for each egg mass. Average egg mass was determined by dividing the egg mass by the number of eggs counted. We used the final mantid weight and the egg mass weight to determine the percent mantid biomass comprised of eggs. The 35-day length of our experiment ensured that all mantids produced a measurable number of eggs but was too short for them to have laid an egg mass. This allowed us to assess how exposure to monarch-sequestered cardenolides affects egg production and reproductive condition. To determine whether mantid mass gain was affected by the type, as opposed to amount, of food consumed, we calculated each mantid’s trophic conversion efficiency as follows: (final − initial mantid biomass)/prey biomass consumed [21].

We also assessed cardenolide levels in *A. curassavica* and the body (i.e., mantid-consumed) tissue of *curassavica*-fed monarch larvae. We first collected fresh leaf (*n* = 10 plants) and caterpillar tissue (*n* = 18), stored it in plastic tubes, and dried it for five days in a 45 °C drying oven. Samples were ground and homogenized following drying, and the powdered tissue was extracted at 2 °C in 95% ethanol at a 1 mL to 100 mg tissue ratio for two days with occasional vortexing; the cardenolide source for the analyses was the 9000× *g* supernatant. We used 3,5-dinitrobenzoic acid (Sigma 121258; [22,23]); in place of 2,2′,4,4′-tetranitrodiphenyl (e.g., [24]). We mixed a 50 µL sample with 50 µL 2% (*w*:*v*) 3,5-dintrobenzoic acid in 100% ethanol and pipetted it into triplicate wells of a Griner UV-Star^®^ 96 well microplate (Sigma-Aldrich, St. Louis, MO, USA). After allowing it to incubate at room temperature for one min, 100 µL 3% NaOH in 100% ethanol was added to each well. The absorbance was quantified at 535 nm after the plate was incubated for ten minutes at room temperature using a Spectramax M2 Multi-Mode spectrophotometer (Molecular Devices, Sunnydale, CA, USA). We corrected for background absorbance using triplicate control wells with 100% ethanol replacing 2% 3,5-dinitrobenzoic acid in 100% ethanol; cardenolide content was expressed as µg digitoxin equivalents per mg dry weight (µg·mg^−1^ DW).

### 2.3. Statistical Analyses

To determine how monarch consumption affected mantid mass gain, we used a MANCOVA to test for among-treatment differences in the amount of prey biomass consumed, trophic conversion efficiency, and mantid final weight. Treatment was the independent variable and mantid initial weight was the covariate. Because the MANCOVA revealed a significant effect of treatment, we ran individual ANCOVAs for each response variable.

To assess how consuming monarch caterpillars affected mantid fecundity, we first ran a MANCOVA on the number of eggs, average egg weight, and percent biomass comprised of eggs. In this analysis, treatment was the independent variable and the covariate was initial live weight. Because the MANCOVA revealed a significant effect of treatment, we analyzed each response variable individually using ANCOVAs. All of the tests met their assumptions, and all analyses were conducted using JMP 9.0 (SAS Institute, Cary, NC, USA).

## 3. Results

*Asclepias currasavica* tissue contained 8.37 ± 0.42 [SE] µg·mg^−1^ DW; the body (i.e., mantid-consumed) tissue of larvae fed on A. currasavica contained lower and more variable but detectable cardenolide levels (6.07 ± 2.11 [SE] µg·mg^−1^ DW).

Mantids accepted both crickets and monarch caterpillars as prey. Some mantids in the low- and medium-toxicity treatments refused to consume monarch caterpillars on the day offered, but accepted them when offered again in subsequent days. Thus, mantids in the low-toxicity treatment each consumed one monarch caterpillar over the 15-day trial period and mantids in the medium-toxicity treatment consumed an average of 4.7 ± 0.18 caterpillars over the 15-day trial period. Mantids in the high-toxicity treatment each consumed 15 caterpillars.

**Figure 1:**
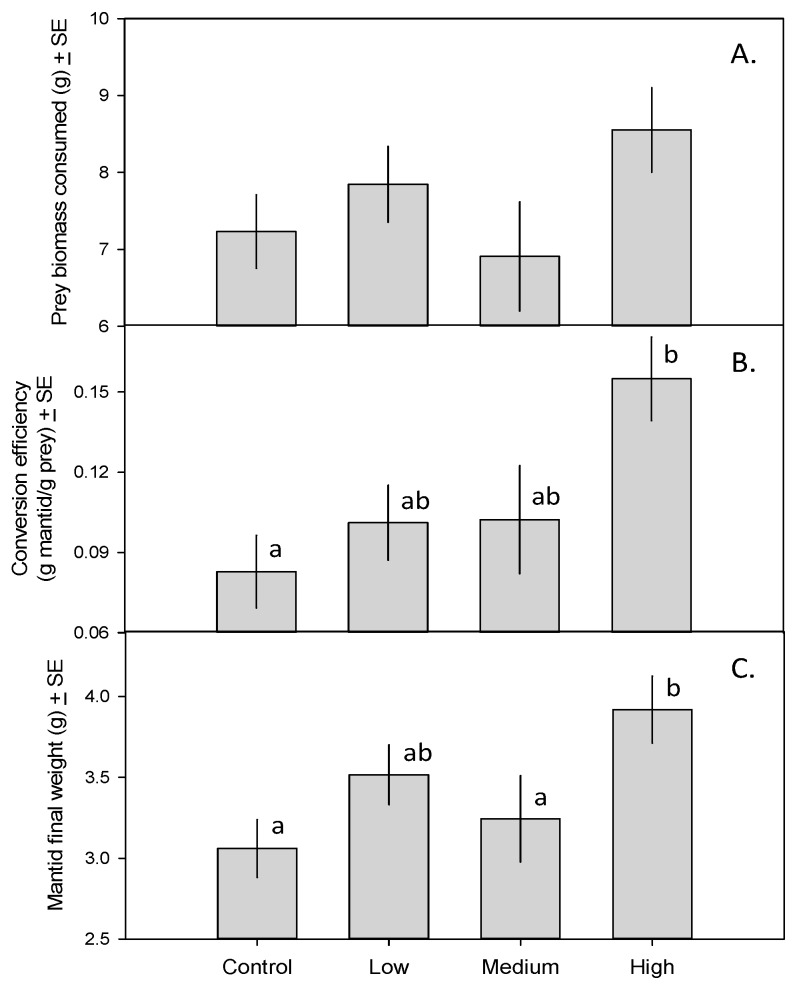
(**A**) Total prey biomass consumed by mantids fed zero, one, five, or 15 monarch caterpillars over a 35-day period ± SE; (**B**) Mantid trophic conversion efficiency (g mantid produced/g prey consumed) ± SE; (**C**) Final mantid weight (g) ± SE. Means with different letters are significantly different.

Mantids in all treatments consumed similar amounts of prey biomass (Figure 1A; *F*_3,23_ = 1.72, *p* = 0.19). Mantids in the high-toxicity treatment (i.e., those eating 15 monarch caterpillars and only crickets thereafter), however, had a substantially higher trophic conversion efficiency (15.6%) than mantids fed only crickets (8.9%; Figure 1B; *F*_3,23_ = 4.01, *p* = 0.020). As a result, mantids in the high-toxicity treatment gained more weight than those in the control and medium-toxicity treatments (Figure 1C; *F*_3,23_ = 4.44, *p* = 0.013).

**Figure 2:**
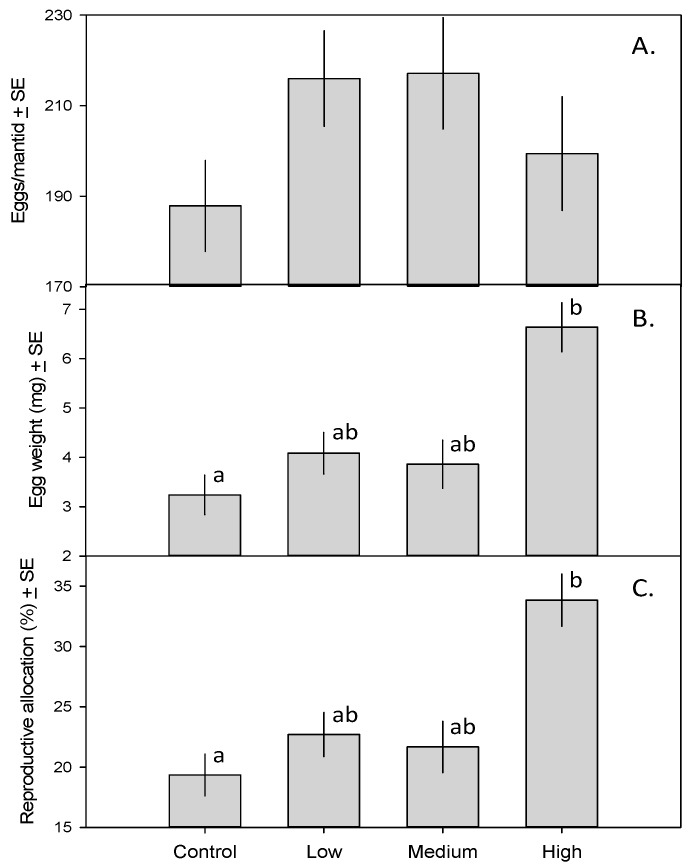
(**A**) Number of eggs produced by mantids fed zero, one, five, or 15 monarch caterpillars ± SE; (**B**) Mean mantid egg weight (mg) ± SE; (**C**) Percent mantid biomass composed of eggs ± SE. Means with different letters are significantly different.

The 31 female mantids in this study produced a total of 6335 eggs. The MANCOVA revealed a significant effect of treatment on mantid fecundity (*F*_9,51_ = 3.53, *p* = 0.002). Although the number of eggs per mantid did not vary across treatments (Figure 2A; *F*_3,23_ = 1.49, *p* = 0.24), eggs of mantids in the high-toxicity treatment weighed more than those of mantids in the control treatment (Figure 2B; *F*_3,23_ = 3.44, *p* = 0.033). As a result, a larger fraction of mantid biomass was devoted to reproduction in the high-toxicity treatment than in the control (Figure 2C; *F*_3,23_ = 3.18, *p* = 0.042).

## 4. Discussion

We did not observe any acute ill-effects of consuming toxic monarch caterpillars on mantids. Per their typical behavior, mantids readily consumed monarch body tissues and rejected the gut material. This behavior and lack of immediate ill-effect is in agreement with our previous work [15,16].

Contrary to our expectations, consuming monarch caterpillars reared on high-cardenolide *Asclepias curassavica* did not reduce mantid fecundity. Instead, mantid egg production was unaffected (Figure 2A) while average egg weight and percent mantid biomass comprised of eggs were both greater in the high toxicity group than in the low and control group (Figure 2B,C, respectively). These data suggest that consumption of monarch prey improves reproductive condition rather than reducing fecundity. It should be noted, however, that our experimental design focused only on egg production as a proxy for reproductive condition; further experimentation might also address egg viability and nymphal survivorship. Also, while we did not measure cardenolide levels in mantid biomass, we did observe that eggs produced by mantids that consumed monarch caterpillars had a green tint. This may be related to the consumption of monarch biomass (hemolymph and other internal fluids of the monarch caterpillar are green).

We initially suspected that the increase in reproductive condition would be explained by differences in consumed prey biomass; this has been shown to affect insect growth, and food-limited adult mantids have lower fecundity [25]. However, our analysis of the total amount of prey biomass consumed revealed no among-treatment differences (Figure 1A). Despite consuming similar amounts of biomass, mantids in the high-toxicity treatment converted a larger fraction of prey biomass into mantid biomass (Figure 1B), and achieved a larger final mass (Figure 1C), than mantids in the control treatment. The increase in reproductive condition (Figure 2) in the high-toxicity treatment could thus be a function of larger body mass and better condition. Chinese mantids lose an average of 47% of their body mass when they oviposit, with larger mantids producing larger ootheca [25]. Another explanation may be an increase in reproductive effort in response to toxicity. In response to parasitism or disease, birds and other species (including invertebrates) may increase investment in reproduction (Reviewed in [26]). Mantids may be similarly responding to cardenolides by increasing their reproductive output.

The among-treatment difference in trophic conversion efficiency has two possible explanations. First, it may be that the nutritional content of even ‘toxic’ monarchs is higher than that of comparably-sized crickets. While monarch caterpillars are soft-bodied, crickets possess a chitinous exoskeleton that may be relatively indigestible; the two organisms may also differ in fat and protein content. Alternatively, the variance in conversion efficiencies could reflect the benefits of diet mixing [27]. While mantids in the toxicity treatments consumed a mixed diet of crickets and mantids, mantids in the control treatment only ate crickets. Mantids fed crickets smeared with pollen have higher reproductive success than mantids fed unsmeared crickets [28] ; similarly, fecundity of the carabid beetle *Agonum dorsale* is highest on a mixed rather than pure diet [29]. Although we had initially conceived the experiment as one in which mantids were fed toxic prey, they may instead have reaped a nutritional benefit through one or both of these mechanisms.

The lack of any negative impact of cardenolides on mantid mass gain and fecundity suggests that mantids are physiologically adapted to tolerate the cardenolide concentrations found in monarch tissues. This is surprising given that late-instar Chinese mantid nymphs quickly learn to avoid consuming cardenolide-sequestering milkweed bugs, *Oncopeltus fasciatus* [30] and early-instar nymphs suffer developmental setbacks associated with consuming diets containing ground *O. fasciatus* or live individuals [17,18]. This may be explained by the fact that while milkweed bugs concentrate cardenolides from their host plant, cardenolide concentrations in monarchs are similar to those found in the host plant [14]. This suggests that higher cardenolide concentrations may indeed be toxic to mantids, and that the ability of mantids to safely consume chemically-defended prey may be determined by toxin concentration. Our experiment reared monarchs on a high-cardenolide host plant (monarchs in our study area typically feed on the relatively low-cardenolide common milkweed, *Asclepias syriaca*), and the high-toxicity treatment offered mantids more monarchs than they would likely encounter in the field. Since our experiment effectively constitutes a ‘worst case scenario’ for mantid exposure to monarch-sequestered cardenolides, the results of our research instead support the hypothesis that monarch-typical cardenolide concentrations pose no threat, and may actually provide nutritional benefits, to Chinese mantids.

## 5. Conclusions

Chinese mantids appear to be able to consume monarch caterpillars without acute ill-effects. Furthermore, despite exposing mantids to atypically high levels of monarch-sequestered cardenolides, we did not observe any negative impacts on fecundity or reproductive condition as measured in this experiment. Instead, our data suggest there may be nutritional benefits that improve reproductive condition. This is likely due to variation in prey nutritional quality and/or diet mixing and is suggestive that mantids are physiologically adapted to incorporate ‘toxic’ monarch caterpillars as part of their diet.

## Figures and Tables

**Table 1:** Description of mantid treatment groups and the number of individuals in each group.

Treatment Group	*n*	Treatment Description
Control	9	Offered two crickets daily from day 4 to day 35
Low Toxicity	8	Offered one monarch caterpillar on day 11. Offered two crickets per day all other days until day 35
Medium Toxicity	7	Offered one monarch on days 6, 9, 12, 15, and 18. Offered two crickets per day all other days
High Toxicity	7	Offered one monarch caterpillar each day on days 4–19. Subsequently, offered two crickets per day until day 35
